# Factors Affecting Survival and Local Control in Patients with Bone Metastases Treated with Radiotherapy

**DOI:** 10.3390/medsci11010017

**Published:** 2023-02-03

**Authors:** Kenji Makita, Yasushi Hamamoto, Hiromitsu Kanzaki, Kei Nagasaki, Noriko Takata, Shintaro Tsuruoka, Kotaro Uwatsu, Teruhito Kido

**Affiliations:** 1 Department of Radiology, Ehime University Graduate School of Medicine, 454 Shitsukawa, Toon 791-0295, Ehime, Japan; 2 Department of Radiation Oncology, National Hospital Organization Shikoku Cancer Center, Kou-160, Minami-Umenomoto-Machi, Matsuyama 791-0280, Ehime, Japan

**Keywords:** bone metastasis, local control, prognosis, external beam radiotherapy, laboratory data

## Abstract

The aim of this study was to evaluate the expected prognosis and factors affecting local control (LC) of the bone metastatic sites treated with palliative external beam radiotherapy (RT). Between December 2010 and April 2019, 420 cases (male/female = 240/180; median age [range]: 66 [12–90] years) with predominantly osteolytic bone metastases received RT and were evaluated. LC was evaluated by follow-up computed tomography (CT) image. Median RT doses (BED_10_) were 39.0 Gy (range, 14.4–71.7 Gy). The 0.5-year overall survival and LC of RT sites were 71% and 84%, respectively. Local recurrence on CT images was observed in 19% (*n* = 80) of the RT sites, and the median recurrence time was 3.5 months (range, 1–106 months). In univariate analysis, abnormal laboratory data before RT (platelet count, serum albumin, total bilirubin, lactate dehydrogenase, or serum calcium level), high-risk primary tumor sites (colorectal, esophageal, hepatobiliary/pancreatic, renal/ureter, and non-epithelial cancers), no antineoplastic agents (ATs) administration after RT, and no bone modifying agents (BMAs) administration after RT were significantly unfavorable factors for both survival and LC of RT sites. Sex (male), performance status (≥3), and RT dose (BED_10_) (<39.0 Gy) were significantly unfavorable factors for only survival, and age (≥70 years) and bone cortex destruction were significantly unfavorable factors for only LC of RT sites. In multivariate analysis, only abnormal laboratory data before RT influenced both unfavorable survival and LC of RT sites. Performance status (≥3), no ATs administration after RT, RT dose (BED_10_) (<39.0 Gy), and sex (male) were significantly unfavorable factors for survival, and primary tumor sites and BMAs administration after RT were significantly unfavorable factors for LC of RT sites. In conclusion, laboratory data before RT was important factor both prognosis and LC of bone metastases treated with palliative RT. At least in patients with abnormal laboratory data before RT, palliative RT seemed to be focused on the only pain relief.

## 1. Introduction

Various tumors frequently have bone metastases, which is the third site for incidence of secondary metastatic lesions, after lung and liver [1,2,3]. While bone metastases often occur following treatment for the primary tumor, they may also present as the initial symptom in up to 20% of cases [4]. The treatment of bone metastases remains controversial for patients with short prognosis expectancy due to advanced cancer, leading to the proposal of non-invasive treatment methods [5].

Radiotherapy is a well-established and non-invasive local treatment modality for bone metastasis. For patients with a short prognosis, it is recommended to use 8 Gy in single fraction (biologically effective dose [BED_10_, BED calculated using an α/β of 10 Gy] = 14.4 Gy) external beam radiotherapy (RT) as palliative treatment to provide pain relief [6,7,8]. For the pain without pathological fractures or spinal cord compression, RT of 8 Gy in single fraction can provide the comparable effect of pain relief to 30 Gy in 10 fraction or 20 Gy in 5 fractions [9]. However, multi-fraction RT (e.g., 10 × 3 Gy [BED_10_ = 39.0 Gy], 5 × 4 Gy [BED_10_ = 28.0 Gy]) has been shown to provide a tendency of a longer duration of pain relief and lower re-treatment rates compared with single-fraction RT, leading to its frequent use in many cases [10,11,12,13]. The difficulty in predicting a prognosis for patients with bone metastases may also contribute to the limited use of single-fraction RT, as patients with a favorable prognosis may require local control (LC) even when pain relief is the primary goal.

Recently, remarkable progress in systemic and supportive therapies has prolonged life expectancy for patients with advanced cancers. Therefore, when palliative RT is used for the pain caused by bone metastases, it will be more important to consider both LC and quality of life when selecting the optimal dose. While many studies have suggested the usefulness of scoring systems for predicting a prognosis in patients with bone metastases [14,15,16,17,18], the relationship between LC of RT sites and the prognosis in patients receiving palliative RT remains unclear. Therefore, in this study, we examined the LC of RT sites and the prognosis in patients with palliative RT to select the optimal radiation method most appropriate for the length of the expected prognosis.

## 2. Materials and Methods

### 2.1. Study Protocol and Lesions

Between January 2010 and December 2019, 2108 bone metastatic sites in 1610 patients were treated with palliative RT in two institutions: National Hospital Organization Shikoku Cancer Center Hospital (*n* = 1514) and Ehime University Hospital (*n* = 594). This study was approved by the Ethics Committee of Ehime University Hospital (registration number: 1912010) and National Hospital Organization Shikoku Cancer Center (registration number: RIN2019-79). The need for informed consent was waived due to the retrospective nature of the study.

**Figure 1:**
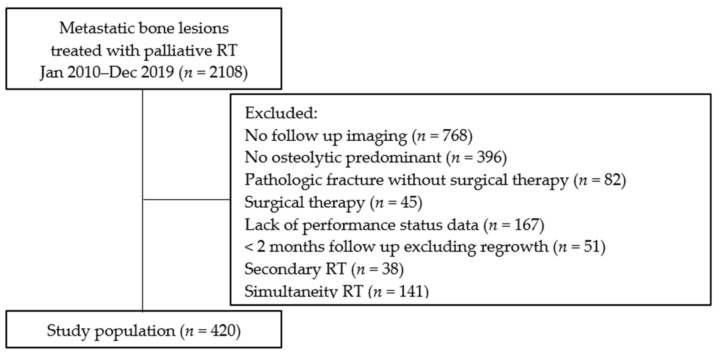
Flow chart. RT, external beam radiotherapy.

The following were the exclusion criteria: absence of follow-up computed tomography (CT) data (*n* = 768), not predominantly osteolytic cancer (*n* = 396), pathologic fracture without surgical treatment (*n* = 82), surgical treatment (*n* = 45), lack of performance status (PS) data (*n* = 167), imaging follow-up time of less than 2 months, excluding regrowth (*n* = 51), and secondary RT (*n* = 38). In addition, when RT was simultaneity performed at ≥2 bone metastatic sites (*n* = 141), LC times were defined as follows: (1) time to local failure when RT sites had local failure or (2) time to longer-term imaging follow-up when RT sites did not have local failure. Thus, we retrospectively evaluated survival and LC of 420 cases (Figure 1).

### 2.2. Classification of Laboratory Data and Primary Tumor Sites

Laboratory data before RT was classified into two groups based on the Katagiri scoring system [14]. Platelet count (<1.0 × 10^5^/μL, *n* = 0), serum albumin (<3.7 g/dL, *n* = 213), total bilirubin (≥1.4 mg/dL, *n* = 15), lactate dehydrogenase (LDH) (≥250 IU/L, *n* = 159), or serum calcium level (≥10.3 mg/dL, *n* = 20) were classified as the abnormal laboratory data. C-reactive proteins (CRP) (≥0.4 mg/dL) were not included because many cases had no CRP data (*n* = 96). Finally, 130 cases had normal laboratory data before RT, and 290 cases had abnormal laboratory data before RT.

The primary tumor sites were classified into two groups based on radiosensitivity and our previous study [19,20]. Colorectal (*n* = 19), esophageal (*n* = 11), hepatobiliary/pancreatic (*n* = 46), renal/ureter (*n* = 37), and non-epithelial (*n* = 13) cancers were classified as the high-risk group (*n* = 126), while the remaining cancers (i.e., head and neck [*n* = 19], breast [*n* = 71], lung [*n* = 146], genitourinary [*n* = 16], and others [*n* = 42]) were classified as the low-risk group (*n* = 294).

### 2.3. Radiotherapy

The gross tumor volume (GTV) was determined by the bone lesions showing osteolytic changes. The clinical target volume (CTV) was determined by expanding 1.0 cm around the GTV in principle. In the vertebral bone metastases, CTV was determined by the entire vertebral bone with GTV. Following that, the planning target volume (PTV) was defined and contoured by expanding 1.0 cm around the CTV in principle. In the vertebral bone metastases, PTV was determined by the upper and lower vertebral body of CTV.

The RT dose was determined at the discretion of each attending physician and institution. The most frequently used RT dose was 30 Gy administered in 10 fractions, which is longer-course RT for the palliation of painful bone metastases [21]. To contrast the various fractionated RT regimens, the biologically effective dose (BED) was calculated. The BED_10_ (BED calculated using an α/β of 10 Gy) was calculated using the equation: *n* × d (1 + d/(α/β)), where d represents the fraction dose, *n* represents the number of fractions, and α/β is 10 Gy.

### 2.4. Evaluation of CT Image

Bone cortex destruction was defined as a break in the continuity of the bone cortex on CT images. The definition of local failure on CT images was enlargement of osteolytic change based on the size prior to RT as a reference. Two observers, blinded to the follow-up information and outcomes, evaluated the CT images.

### 2.5. Statistical Analysis

The time of survival and LC of the RT sites were compared using the Kaplan–Meier method. Cox proportional hazards models were used to determine hazard ratio (HR), including 95% confidence interval (CI), and *p*-value. Univariate and multivariate analyses were performed to assess the predictive factors associated with overall survival (OS) and LC rates of RT sites. Factors included in the multivariate analysis had a *p*-value of <0.05 in the univariate analysis. In the multivariate analysis, statistical significance was defined as *p*-value < 0.05. All statistical analyses were performed using JMP software (JMP version 14.3.0; SAS Institute, Cary, NC, USA).

## 3. Results

The characteristics of all the cases were presented in Table 1. A total of 420 patients (male/female = 240/180; median age [range]: 66 [12–90] years) were included in the analysis. The median follow-up and imaging follow-up times were 11 months (range, 1–115 months) and 7 months (range, 1–111 months), respectively. There were 230 lesions of vertebrae, 99 lesions of the pelvis, 33 lesions of ribs, and 58 lesions of other bone metastases treated with palliative RT. In addition, there were 310 bone metastatic sites with bone cortex destruction. The median RT dose (BED_10_) was 30 Gy in 10 fractions (39.0 Gy). The other fraction schedules, in sequential order, were as follows: 1 × 8 Gy (14.4 Gy), 5 × 4 Gy (28.0 Gy), 10 × 2.5 Gy (31.2 Gy), 15–16 × 2.5 Gy (46.9–50.0 Gy), 12–15 × 3 Gy (46.8–58.5 Gy), 25 × 2 Gy (60.0 Gy), 5 × 4 Gy + 3 × 3 Gy (39.7 Gy), and 3 × 3 Gy + 25 × 2 Gy (71.7 Gy).

### 3.1. The Factors Affecting Survival Rates

**Figure 2:**
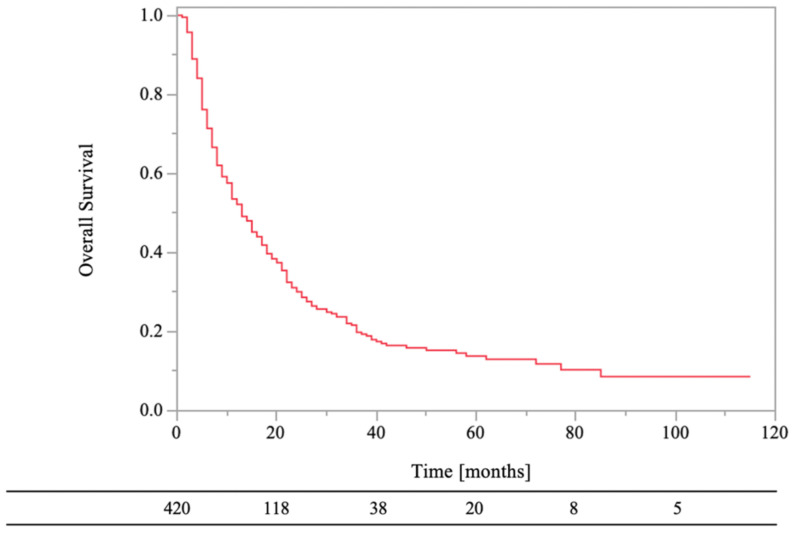
Overall survival of all patients.

The 0.5- and 1-year OS rates were 71% and 52%, respectively (Figure 2). In the univariate analyses, there were statistically significant differences in survival rates between female and male (HR, 1.50; 95% CI, 1.19–1.89; *p* < 0.01), PS < 3 and PS ≥ 3 (HR, 1.38; 95% CI, 1.05–1.81; *p* = 0.02), normal laboratory data before RT and abnormal laboratory data before RT (HR, 1.62; 95% CI, 1.26–2.07; *p* < 0.01), low-risk primary tumor sites and high-risk primary tumor sites (HR, 1.43; 95% CI, 1.11–1.84; *p* < 0.01), RT dose (BED_10_) of ≥39.0 Gy and RT dose (BED_10_) of <39.0 Gy (HR, 2.30; 95% CI, 1.62–3.27; *p* < 0.01), antineoplastic agents (aTs) after RT and no aTs after RT (HR, 1.69; 95% CI, 1.31–2.18; *p* < 0.01), and bone modifying agents (BMAs) after RT and no BMAs after RT (HR, 1.48; 95% CI, 1.17–1.88; *p* < 0.01). On the other hand, there were not statistically significant differences in survival rates between age of <70 years and age of ≥70 years (HR, 1.05; 95% CI, 0.83–1.33; *p* = 0.68), bone cortex destruction and no bone cortex destruction (HR, 0.95; 95% CI, 0.73–1.23; *p* = 0.68), and aTs before RT and no aTs before RT (HR, 0.90; 95% CI, 0.72–1.14; *p* = 0.39).

In the multivariate analyses, male (HR, 1.38; 95%CI, 1.09–1.76; *p* < 0.01), PS ≥ 3 (HR, 1.38; 95% CI, 1.04–1.82; *p* = 0.03), abnormal laboratory data before RT (HR, 1.59; 95% CI, 1.23–2.04; *p* < 0.01), RT dose (BED_10_) <39.0 Gy (HR, 2.15; 95% CI, 1.50–3.07; *p* < 0.01), and no aTs after RT (HR, 1.57; 95% CI, 1.20–2.05; *p* < 0.01) were significantly unfavorable factors for survival rates (Table 2). High-risk primary tumor sites (HR, 1.28; 95% CI, 0.99–1.66; *p* = 0.06) and no BMAs after RT (HR, 1.29; 95% CI, 1.00–1.65; *p* = 0.05) tended to be significantly unfavorable factors for survival rates.

### 3.2. The Factors Affecting LC Rates

**Figure 3:**
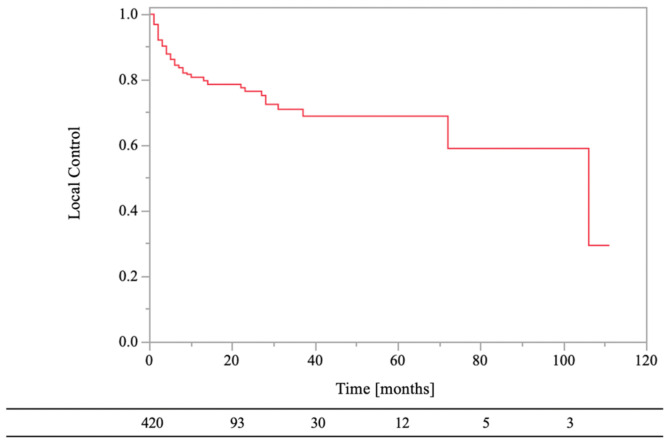
Local control of bone metastases.

The 0.5- and 1-year LC rates of the RT sites were 84% and 81%, respectively (Figure 3). Local recurrence on CT images was observed in 19% (*n* = 80) of the lesions, and the median recurrence time was 3.5 months (range, 1–106 months). In the univariate analyses, there were statistically significant differences in LC of RT sites between age of <70 years and age of ≥70 years (HR, 1.79; 95% CI, 1.15–2.79; *p* = 0.01), normal laboratory data before RT and abnormal laboratory data before RT (HR, 2.07; 95% CI, 1.22–3.51; *p* = 0.01), low-risk primary tumor sites and high-risk primary tumor sites (HR, 4.34; 95% CI, 2.78–6.78; *p* < 0.01), bone cortex destruction and no bone cortex destruction (HR, 0.54; 95% CI, 0.30–0.98; *p* = 0.04), aTs after RT and no aTs after RT (HR, 2.54; 95%CI, 1.61–4.02; *p* < 0.01), and BMAs after RT and no BMAs after RT (HR, 2.35; 95% CI, 1.51–3.67; *p* < 0.01). On the other hand, there were not statistically significant differences in LC of RT sites between female and male (HR, 1.59; 95% CI, 1.00–2.51; *p* = 0.05), PS < 3 and PS ≥ 3 (HR, 1.06; 95% CI, 0.58–1.92; *p* = 0.85), RT dose (BED_10_) of ≥39.0 Gy and RT dose (BED_10_) of <39.0 Gy (HR, 1.67; 95% CI, 0.82–3.34; *p* = 0.16), and aTs before RT and no aTs before RT (HR, 0.98; 95% CI, 0.63–1.53; *p* = 0.94).

In the multivariate analyses, abnormal laboratory data before RT (HR, 2.16; 95% CI, 1.26–3.73; *p* < 0.01), high-risk primary tumor sites (HR, 4.15; 95% CI, 2.64–6.52; *p* < 0.01), and no BMAs after RT (HR, 1.85; 95% CI, 1.15–2.98; *p* = 0.01) were significantly unfavorable factors for LC of RT sites (Table 3). Age of ≥70 years (HR, 1.52; 95% CI, 0.96–2.39; *p* = 0.07) and no aTs after RT (HR, 1.63; 95% CI, 0.98–2.71; *p* = 0.06) tended to be significantly unfavorable factors for LC of RT sites.

## 4. Discussion

This study showed that the 0.5-year OS rates and LC of RT sites were approximately 70% and 80% in patients who needed palliative RT for bone metastases. Only laboratory data before RT influenced both survival and LC of RT sites. Sex, PS, RT dose (BED_10_), and aTs after RT were significant factors for only survival rates for bone metastatic patients treated with palliative RT. In addition, primary tumor sites and BMAs after RT were significant factors for only LC rates for bone metastatic sites treated with palliative RT.

In our study, only laboratory data before RT had a large impact for both survival and LC in patients with palliative RT. Many studies showed that laboratory data were important for prediction of survival [14,22,23,24,25]. These laboratory data are considered to represent the tumor aggressiveness. In our study, inflammatory response markers (neutrophil to lymphocyte ratio, platelet to lymphocyte ratio, or C-reactive protein albumin ratio) were not measured because many patients were treated with best supportive care. Therefore, laboratory data on predictive prognosis and LC of RT sites were referenced the laboratory data studied by Katagiri et al. [14]. Because these abnormal laboratory data could be an indicator of disease aggressiveness and lead both unfavorable prognosis and unfavorable LC of RT sites, the use of 8 Gy of single-fraction RT could be more recommended. On the other hand, although PS ≥ 3 as a general condition including tumor aggressiveness was a significantly unfavorable factor for prognosis, it was not a significantly unfavorable factor for LC of RT sites. One of the possible reasons for this is that PS is influenced by the patient’s age and the evaluation of each oncologist. Therefore, although PS was considered important as a predictor of prognosis, it was inadequate as a predictor of LC of RT sites.

Primary tumor sites were significantly important factor for the LC of RT sites, and it also tended to affect survival rates. In our study, because the classification of primary tumor sites was focused on LC of RT sites, the difference of survival rates was smaller than if it was focused on survival rates. Many studies have suggested prognostic differences by primary tumor site [14,15,26]. Although the primary tumor sites should be evaluated as factors affecting both prognosis and LC of RT sites in clinical practice, the classification of the primary tumor sites as predictors of prognosis needs to be different from the classification used in our study.

Systemic therapies (ATs and BMAs) after RT were important for prognosis and LC of RT sites. Administration of ATs after RT was more important for the prognosis, and administration of BMAs after RT was more important for the LC of RT sites. Many studies showed that ATs influenced the prognosis and LC of bone metastases and BMAs influenced the LC of bone metastases [27,28,29,30]. These systemic therapy administrations were important in predicting prognosis and determining RT dose. When these systemic therapies were administered for the patients with bone metastases, LC of RT sites was not unfavorable regardless of palliative RT dose. Although further studies were warrened because of the large number of censored cases with RT dose (BED_10_) < 39.0 Gy in our study, 8 Gy in single fraction may be a treatment option in terms of LC of bone metastases.

RT dose (BED_10_) did not significantly correlate with the LC of RT sites in contrast to previous studies [19,31,32]. One of possible explanation for this was that the impact of RT for LC may be masked by the primary tumor sites, abnormal laboratory data before RT, and BMAs after RT, which had higher impact for LC of bone metastases. When the primary tumor sites were classified in the low-risk group (e.g., lung or breast cancer), the lower RT dose for bone metastases may have potential to achieve adequate LC compared to higher RT doses. On the other hand, in our study, RT dose (BED_10_) significantly correlated with the survival rates. Although RT for bone metastases is known to contribute to pain relief and improvement of quality of life, it does not contribute to prolonging prognosis [9,33]. Therefore, this indicated that the patient’s prognosis predicted by each radiation oncologists and institution in clinical practice was acceptable enough. In addition, although sex correlated with the survival rates in our study, this may be due to excluding all osteoblastic bone metastases including prostate cancer. Prostate cancer has a good prognosis compared to many other tumors [14]. On the other hand, breast cancer, which is most common tumor in females and has a good prognosis, was included in our study. Therefore, the exclusion of osteoblastic bone metastases may have influenced the difference in prognosis according to sex.

## 5. Limitations

This study had several limitations because of its retrospective design. Firstly, this study was affected by the small sample size and heterogenous of evaluated population. Therefore, cautious interpretation of these findings is imperative. Secondly, many insufficient data on many prognostic factors (the number of bone metastases, the major internal organs metastases, and CRP values) were influenced the statistical analyses. However, these data could not be obtained in some cases in clinical practice. Therefore, we must predict the prognosis based on the little information and select the appropriate treatment for the patients with bone metastases. Many studies have investigated prognosis and pain relief, and have not focused on LC of RT sites in patient with palliative intent RT. Although future studies are necessary to verify these findings, the novelty of this study is that we investigated the correlation between prognosis and LC of RT sites, and this study provides an important consideration for selecting palliative RT doses for bone metastases.

## 6. Conclusions

Laboratory data before RT significantly correlated to both prognosis and LC of RT sites in bone metastatic patients. At least in patients with abnormal laboratory data before RT, palliative RT seemed to be focused only on the pain relief.

## Figures and Tables

**Table 1:** Characteristics.

Factors		No. of Cases	%
age	<70 years	275	65.5
	≥70 years	145	34.5
sex	male	240	57.1
	female	180	42.9
ECOG PS	<3	341	81.2
	≥3	79	18.8
laboratory data before RT	normal	130	31.0
	abnormal	290	60.0
primary tumor sites	lung	146	34.8
	breast	71	16.9
	head and neck	19	4.5
	esophagus	11	2.6
	hepatobiliary/pancreatic	46	11.0
	renal/ureter	37	8.8
	colorectal	19	4.5
	gynecological	16	3.8
	non-epithelial	13	3.1
	others	42	10.0
RT sites	vertebral	230	54.8
	pelvis	99	23.6
	rib	33	7.9
	others	58	13.8
RT dose (BED_10_)	median: 39.0 Gy (14.4–71.7 Gy)		
	14.4 Gy	7	1.7
	>14.4 Gy, <39.0 Gy	36	8.6
	39.0 Gy	261	62.1
	>39.0 Gy	116	27.6
bone cortex destruction	yes	310	73.8
	no	110	26.2
aTs before RT	yes	232	55.2
	no	188	44.8
aTs after RT	yes	301	71.7
	no	119	28.3
BMAs after RT	yes	276	65.7
	no	144	34.3

ECOG PS, Eastern Cooperative Oncology Group Performance Status; RT, external beam radiotherapy; BED, biologically effective dose; aTs, antineoplastic agents; BMAs, bone modifying agents.

**Table 2:** Results of univariate and multivariate analyses for survival rates after RT.

		0.5-Year (%)	1-Year (%)	Univariate Analysis		Multivariate Analysis	
				HR (95% CI)	*p*	HR (95% CI)	*p*
age	<70 years vs. ≥70 years	72 vs. 71	53 vs. 52	1.05 (0.83–1.33)	0.68	-	-
sex	female vs. male	82 vs. 63	64 vs. 43	1.50 (1.19–1.89)	<0.01	1.38 (1.09–1.76)	<0.01
ECOG PS	<3 vs. ≥3	74 vs. 59	55 vs. 38	1.38 (1.05–1.81)	0.02	1.38 (1.04–1.82)	0.03
laboratory data before RT	normal vs. abnormal	89 vs. 63	68 vs. 45	1.62 (1.26–2.07)	<0.01	1.59 (1.23–2.04)	<0.01
primary tumor sites	low risk vs. high risk	74 vs. 64	56 vs. 42	1.43 (1.11–1.84)	<0.01	1.28 (0.99–1.66)	0.06
bone cortex destruction	yes vs. no	72 vs. 71	51 vs. 55	0.95 (0.73–1.23)	0.68	-	-
RT dose (BED_10_)	≥39.0 Gy vs. <39.0 Gy	75 vs. 37	56 vs. 21	2.30 (1.62–3.27)	<0.01	2.15 (1.50–3.07)	<0.01
aTs before RT	yes vs. no	70 vs. 73	51 vs. 53	0.90 (0.72–1.14)	0.39	-	-
aTs after RT	yes vs. no	77 vs. 56	57 vs. 40	1.69 (1.31–2.18)	<0.01	1.57 (1.20–2.05)	<0.01
BMAs after RT	yes vs. no	77 vs. 61	57 vs. 43	1.48 (1.17–1.88)	<0.01	1.29 (1.00–1.65)	0.05

ECOG PS, Eastern Cooperative Oncology Group Performance Status; RT, external beam radiotherapy; BED, biologically effective dose; aTs, antineoplastic agents; BMAs, bone modifying agents.

**Table 3:** Results of univariate and multivariate analyses for local control of RT sites.

		0.5-Year (%)	1-Year (%)	Univariate Analysis		Multivariate Analysis	
				HR (95% CI)	*p*	HR (95% CI)	*p*
age	<70 years vs. ≥70 years	89 vs. 77	84 vs. 74	1.79 (1.15–2.79)	0.01	1.52 (0.96–2.39)	0.07
sex	female vs. male	88 vs. 81	85 vs. 77	1.59 (1.00–2.51)	0.05	-	-
ECOG PS	<3 vs. ≥3	85 vs. 83	81 vs. 81	1.06 (0.58–1.92)	0.85	-	-
laboratory data before RT	normal vs. abnormal	94 vs. 80	90 vs. 76	2.07 (1.22–3.51)	0.01	2.16 (1.26–3.73)	<0.01
primary tumor sites	low risk vs. high risk	90 vs. 70	89 vs. 56	4.34 (2.78–6.78)	<0.01	4.15 (2.64–6.52)	<0.01
bone cortex destruction	yes vs. no	81 vs. 94	79 vs. 86	0.54 (0.30–0.98)	0.04	0.59 (0.33–1.08)	0.09
RT dose (BED_10_)	≥39.0 Gy vs. <39.0 Gy	85 vs. 75	82 vs. 66	1.67 (0.82–3.34)	0.16	-	-
aTs before RT	yes vs. no	85 vs. 83	81 vs. 81	0.98 (0.63–1.53)	0.94	-	-
aTs after RT	yes vs. no	89 vs. 70	86 vs. 64	2.54 (1.61–4.02)	<0.01	1.63 (0.98–2.71)	0.06
BMAs after RT	yes vs. no	89 vs. 76	86 vs. 70	2.35 (1.51–3.67)	<0.01	1.85 (1.15–2.98)	0.01

ECOG PS, Eastern Cooperative Oncology Group Performance Status; RT, external-beam radiotherapy; BED, biologically effective dose; aTs, antineoplastic agents; BMAs, bone modifying agents.

## Data Availability

Not applicable.

## References

[1] Nystrom J.S., Weiner J.M., Heffelfinger-Juttner J., Irwin L.E., Bateman J.R., Wolf R.M. (1977). Metastatic and histologic presentations in unknown primary cancer. Semin. Oncol..

[2] Greco T., Fulchignoni C., Cianni L., Maccauro G., Perisano C. (2022). Surgical management of tibial metastases: A systematic review. Acta Biomed..

[3] Coleman R.E. (2001). Metastatic bone disease: Clinical features, pathophysiology and treatment strategies. Cancer Treat. Rev..

[4] De Felice F., Piccioli A., Musio D., Tombolini V. (2017). The role of radiation therapy in bone metastases management. Oncotarget.

[5] Perisano C., Greco T., Fulchignoni C., Maccauro G., Perisano C. (2022). The IlluminOss(R) System: A solution in elderly patients with upper limbs bone metastases. Eur. Rev. Med. Pharmacol. Sci..

[6] Lutz S., Balboni T., Jones J., Lo S., Petit J., Rich S.E., Wong R., Hahn C. (2017). Palliative radiation therapy for bone metastases: Update of an ASTRO Evidence-Based Guideline. Pract. Radiat. Oncol..

[7] Oldenburger E., Brown S., Willmann J., van der Velden J.M., Spalek M., van der Linden Y.M., Kazmierska J., Menten J., Andratschke N., Hoskin P. (2022). ESTRO ACROP guidelines for external beam radiotherapy of patients with complicated bone metastases. Radiother. Oncol..

[8] Van der Velden J., Willmann J., Spalek M., Oldenburger E., Brown S., Kazmierska J., Andratschke N., Menten J., van der Linden Y., Hoskin P. (2022). ESTRO ACROP guidelines for external beam radiotherapy of patients with uncomplicated bone metastases. Radiother. Oncol..

[9] Chow E., Zeng L., Salvo N., Dennis K., Tsao M., Lutz S. (2012). Update on the systematic review of palliative radiotherapy trials for bone metastasis. Clin. Oncol..

[10] Olson R.A., Tiwana M.S., Barnes M., Kiraly A., Beecham K., Miller S., Hoegler D., Olivotto I. (2014). Use of single- versus multiple-fraction palliative radiation therapy for bone metastases: Population-based analysis of 16,898 courses in a Canadian province. Int. J. Radiat. Oncol. Biol. Phys..

[11] Logan J.K., Jiang J., Shih Y.T., Lei X., Xu Y., Hoffman K.E., Giordano S.H., Smith B.D. (2019). Trends in Radiation for Bone Metastasis During a Period of Multiple National Quality Improvement Initiatives. J. Oncol. Pract..

[12] Steenland E., Leer J.W., van Houwelingen H., Post W.J., van den Hout W.B., Kievit J., de Haes H., Martijn H., Oei B., Vonk E. (1999). The effect of a single fraction compared to multiple fractions on painful bone metastases: A global analysis of the Dutch Bone Metastasis Study. Radiother. Oncol..

[13] Rich S.E., Chow R., Raman S., Liang Zeng K., Lutz S., Lam H., Silva M.F., Chow E. (2018). Update of the systematic review of palliative radiation therapy fractionation for bone metastases. Radiother. Oncol..

[14] Katagiri H., Okada R., Takagi T., Takahashi M., Murata H., Harada H., Nishimura T., Asakura H., Ogawa H. (2014). New prognostic factors and scoring system for patients with skeletal metastasis. Cancer Med..

[15] Tokuhashi Y., Matsuzaki H., Oda H., Oshima M., Ryu J. (2005). A revised scoring system for preoperative evaluation of metastatic spine tumor prognosis. Spine.

[16] Bollen L., van der Linden Y.M., Pondaag W., Fiocco M., Pattynama B.P., Marijnen C.A., Nelissen R.G., Peul W.C., Dijkstra P.D. (2014). Prognostic factors associated with survival in patients with symptomatic spinal bone metastases: A retrospective cohort study of 1043 patients. Neuro Oncol..

[17] Westhoff P.G., de Graeff A., Monninkhof E.M., Bollen L., Dijkstra S.P., van der Steen-Banasik E.M., van Vulpen M., Leer J.W., Marijnen C.A., van der Linden Y.M. (2014). An easy tool to predict survival in patients receiving radiation therapy for painful bone metastases. Int. J. Radiat. Oncol. Biol. Phys..

[18] Willeumier J.J., van der Linden Y.M., van der Wal C.W.P.G., Jutte P.C., van der Velden J.M., Smolle M.A., van der Zwaal P., Koper P., Bakri L., de Pree I. (2018). An easy-to-use prognostic model for survival estimation for patients with symptomatic long bone metastases. J. Bone Jt. Surg. Am..

[19] Makita K., Hamamoto Y., Kanzaki H., Kataoka M., Yamamoto S., Nagasaki K., Ishikawa H., Takata N., Tsuruoka S., Uwatsu K. (2021). Local control of bone metastases treated with external beam radiotherapy in recent years: A multicenter retrospective study. Radiat. Oncol..

[20] Gerszten P.C., Mendel E., Yamada Y. (2009). Radiotherapy and radiosurgery for meta-static spine disease: What are the options, indications, and outcomes?. Spine.

[21] Hartsell W.F., Scott C.B., Bruner D.W., Scarantino C.W., Ivker R.A., Roach M., Suh J.H., Demas W.F., Movsas B., Petersen I.A. (2005). Randomized trial of short versus long-course radiotherapy for palliation of painful bone metastases. J. Natl. Cancer Inst..

[22] Takenaka Y., Oya R., Kitamiura T., Ashida N., Shimizu K., Takemura K., Yamamoto Y., Uno A. (2018). Prognostic role of neutrophil-to-lymphocyte ratio in head and neck cancer: A meta-analysis. Head Neck.

[23] Zhou X., Du Y., Huang Z., Xu J., Qiu T., Wang J., Wang T., Zhu W., Liu P. (2014). Prognostic value of PLR in various cancers: A meta-analysis. PLoS ONE.

[24] Yen H.K., Yen H.K., Hu M.H., Zijlstra H., Groot O.Q., Hsieh H.C., Yang J.J., Karhade A.V., Chen P.C., Chen Y.H. (2022). Prognostic significance of lab data and performance comparison by validating survival prediction models for patients with spinal metastases after radiotherapy. Radiother. Oncol..

[25] Makita K., Hamamoto Y., Takata N., Ishikawa H., Tsuruoka S., Uwatsu K., Hato N., Kido T. (2021). Prognostic significance of inflammatory response markers for locally advanced squamous cell carcinoma of the external auditory canal and middle ear. J. Radiat. Res..

[26] Leithner A., Radl R., Gruber G., Hochegger M., Leithner K., Welkerling H., Rehak P., Windhager R. (2008). Predictive value of seven preoperative prognostic scoring systems for spinal metastases. Eur. Spine J..

[27] Ciray I., Lindman H., Aström K.G. (2001). Early response of breast cancer bone metastases to chemotherapy evaluated with MR imaging. Acta Radiol..

[28] Tokito T., Shukuya T., Akamatsu H., Taira T., Ono A., Kenmotsu H., Naito T., Murakami H., Takahashi T., Endo M. (2013). Efficacy of bevacizumab-containing chemotherapy for non-squamous non-small cell lung cancer with bone metastases. Cancer Chemother. Pharmacol..

[29] Rades D., Hakim S.G., Bajrovic A., Karstens J.H., Veninga T., Rudat V., Schild S.E. (2012). Impact of zoledronic acid on control of metastatic spinal cord compression. Strahlenther. Onkol..

[30] Vassiliou V., Kalogeropoulou C., Christopoulos C., Solomou E., Leotsinides M., Kardamakis D. (2007). Combination ibandronate and radiotherapy for the treatment of bone metastases: Clinical evaluation and radiologic assessment. Int. J. Radiat. Oncol. Biol. Phys..

[31] Makita K., Hamamoto Y., Tsuruoka S., Takata N., Urashima Y., Miyagawa M., Mochizuki T. (2020). Treatment intensity and control rates in combining external-beam radiotherapy and radioactive iodine therapy for metastatic or recurrent differentiated thyroid cancer. Int. J. Clin. Oncol..

[32] Rades D., Panzner A., Rudat V., Karstens J.H., Schild S.E. (2011). Dose escalation of radiotherapy for metastatic spinal cord compression (MSCC) in patients with relatively favorable survival prognosis. Strahlenther. Onkol..

[33] Li K.K., Chow E., Chiu H., Bradley N., Doyle M., Barnes E.A., Tsao M., Sinclair E., Danjoux C. (2006). Effectiveness of palliative Radiotherapy in the Treatment of Bone Metastases Employing the Brief Pain Inventory. J. Cancer Pain Symptom Palliation.

